# High-Precision Distribution of Highly Stable Optical Pulse Trains with 8.8 × 10^−19^ instability

**DOI:** 10.1038/srep05109

**Published:** 2014-05-29

**Authors:** B. Ning, S. Y. Zhang, D. Hou, J. T. Wu, Z. B. Li, J. Y. Zhao

**Affiliations:** 1 Department of Electronics, Peking University, Beijing 100871, China, State Key Laboratory of Advanced Optical Communication Systems and Networks, Peking University, Beijing 100871, China

## Abstract

The high-precision distribution of optical pulse trains via fibre links has had a considerable impact in many fields. In most published work, the accuracy is still fundamentally limited by unavoidable noise sources, such as thermal and shot noise from conventional photodiodes and thermal noise from mixers. Here, we demonstrate a new high-precision timing distribution system that uses a highly precise phase detector to obviously reduce the effect of these limitations. Instead of using photodiodes and microwave mixers, we use several fibre Sagnac-loop-based optical-microwave phase detectors (OM-PDs) to achieve optical-electrical conversion and phase measurements, thereby suppressing the sources of noise and achieving ultra-high accuracy. The results of a distribution experiment using a 10-km fibre link indicate that our system exhibits a residual instability of 2.0 × 10^−15^ at1 s and8.8 × 10^−19^ at 40,000 s and an integrated timing jitter as low as 3.8 fs in a bandwidth of 1 Hz to 100 kHz. This low instability and timing jitter make it possible for our system to be used in the distribution of optical-clock signals or in applications that require extremely accurate frequency/time synchronisation.

The distribution of high-precision timing signals (*e.g.*, microwaves or optical pulses) is required for many endeavours, ranging from practical applications to scientific research. In fact, these techniques provide the direct basis of many applications (*e.g.*, in telecommunication, navigation, astronomy and physical experiments^1^,^2^,^3^,^4^,^5^,^6^,^7^,^8^). Usually, the most precise reference sources are only operated at specialised laboratories, and sharing these reference signals typically requires high-precision techniques for distributing timing signals. Especially in some modern large-scale scientific facilities, such as X-ray free-electron lasers (XFELs) and particle accelerators, the distribution must retain extremely high timing accuracy to synchronise the source with remotely located sites^2^,^9^,^10^,^11^. Over the past few decades, a variety of RF signal-distribution techniques have been developed. One commonly employed approach involves satellites; this approach serves as the basis for such methods as Global Positioning System and two-way satellite time and frequency transfer^12^,^13^.

Satellite methods, however, offer only moderate accuracy and are unsuitable for most of the aforementioned modern applications. For such applications, the current most appropriate and elegant method involves the use of optical-fibre links to distribute the RF signals^3^. Research has demonstrated that fibre-based techniques are orders of magnitude more accurate and more stable than satellite methods^3^, as fibre possesses both low attenuation and high reliability. For fibre-based techniques, lasers play a very crucial role because they are used as the transmitting sources and directly determine the distribution performance. State-of-the-art fibre-based RF signal-distribution techniques can provide stabilities of 10^−19^ over duration of one day^14^. Although RF transfer via modulated continuous-wave lasers is a very direct method^14^,^15^,^16^,^17^, the use of mode-locked lasers (MLLs) as sources has recently attracted more interest^2^,^18^,^19^,^20^,^21^,^22^. An MLL can simultaneously provide optical and microwave timing information. With careful optimisation^23^,^24^, MLLs can easily generate regularly spaced optical pulse trains with extremely low (*i.e.*, attosecond-scale) timing jitter, which allows the timing signals to be distributed with unprecedented precision^2^. Many studies have already been conducted in this area; however, much of this work has involved the use of photodiodes and microwave mixers for optical-electrical conversion and phase measurements in certain necessary procedures^14^,^15^,^16^,^17^,^18^,^19^,^20^,^21^,^22^, including microwave-to-optic modulation, fibre-link stabilisation and RF signal extraction. Unfortunately, photodiodes and microwave mixers can introduce unavoidable timing noise (*e.g.*, shot noise, thermal noise and amplitude-modulation to phase-modulation (AM-to-PM) noise^25^,^26^,^27^). These noise sources reduce the precision of the laser and limit the distribution accuracy to the scale of tens of femtoseconds (typically >15 fs for timing jitter and >30 fs for long-term drift).

In this paper, we demonstrate a new fibre-based distribution system for optical pulse signals. Instead of using traditional electronic devices, we utilise some easily implemented fibre Sagnac-loop-based optical-microwave phase detectors (OM-PDs) to perform all the aforementioned optics- and microwave-related procedures with timing accuracies of several hundred attoseconds. We test our system on a 10-km outdoor fibre link, and the results indicate that the distribution precision overcame the limitation of conventional RF transfer^14^,^15^,^16^,^17^,^18^,^19^,^20^,^21^,^22^ by achieving a 3.8-fs integrated timing jitter in a bandwidth of 1 Hz to 100 kHz and a 22-fs root-mean-square (rms) timing drift over 72 hours. The residual instability reached the 8.8 × 10^−19^ level at 40,000 s averaging times, which is quite sufficient for the long-term transfer of the majority of advanced clock standards, such as optical clocks. Therefore, our frequency-distribution technique may provide a very powerful tool for transferring the timing signals of optical clocks without loss of stability. This technique can also be used in the application of facilities which necessarily require high-precision and long-term synchronisation, such as the next-generation of X-ray free-electron lasers.

## Results

### High-precision stabilisation of a femtosecond MLL

**Figure 1:**
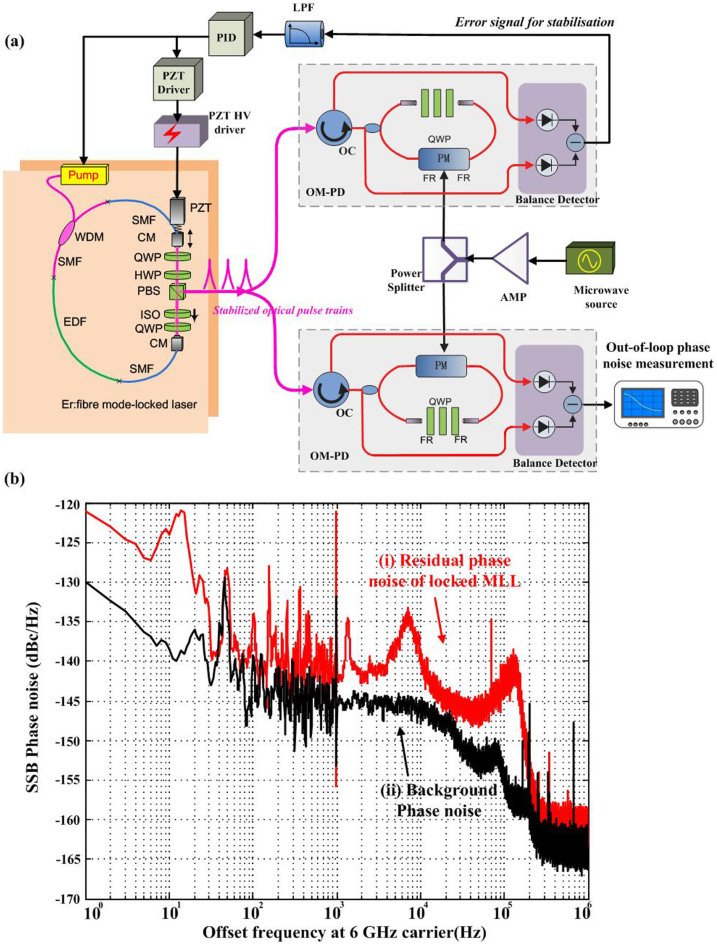
High-precision stabilisation of an MLL and the measured residual phase noise. (a) Schematic of the high-precision stabilisation of an MLL achieved by locking the laser to a microwave reference via an OM-PD. HV: high voltage; PID: proportional-integral-derivative controller; LPF: low-pass filter; PZT: piezoelectric transducer; CM: collimator; HWP: half-wave plate; QWP: quarter-wave plate; SMF: single-mode fibre; EDF: erbium-doped fibre; PBS: polarisation beam splitter; WDM: wavelength division multiplexer; Amp: amplifier; FR: faraday rotator; OC: optical circulator; PM: phase modulator. (b) Measured residual phase noise of the phase locking. The locking bandwidth is set to 100 kHz. (i) Out-of-loop residual phase noise of MLL locked to a RF reference. (ii) Background noise of the OM-PD.

Figure 1a outlines the principles of the high-precision stabilisation of an MLL achieved by locking the laser to a microwave reference via an OM-PD. Stabilisation of an MLL is achieved by phase locking the laser to a stable microwave reference with ultra-high accuracy. Conventionally, the phase locking is achieved by first detecting the phase of the MLL using a photodiode, comparing the phases of the MLL and the reference microwave using a frequency mixer and finally, using the phase-error signal to adjust the length of an intra-cavity piezoelectric transducer (PZT), thereby controlling the phase of the MLL. This technique, however, suffers from shot and thermal noise generated by the photodiode and thermal noise generated by the frequency mixers; it is also limited by the resolution and response time of the PZT. As a result, the accuracy of the stabilisation is limited to tens of femtoseconds. To overcome these limitations, we remove the photodiode and frequency mixer and use an OM-PD for the phase comparison. Moreover, to control the phase of the MLL much more accurately, we introduce a new technique. Both pump modulation and PZT control are applied to ensure long-term, high-accuracy control of the laser phase^28^. More information concerning this technique can be found in Ref. 28 (also see the Methods section).

Using the new stabilisation technique for the MLL, we can successfully phase lock the MLL to a 6-GHz reference microwave signal. The residual phase noise^29^,^30^ of the phase locking is measured to evaluate the performance. Figure 1b presents the measured data. The phase noise reaches-121 dBc/Hz and -145 dBc/Hz at 1 Hz and 100 kHz offset frequency, respectively. By integrating the phase noise in a bandwidth of 1 Hz to 100 kHz, we find an integrated timing jitter of 1.2 fs. With the combination of pump modulation and PZT control and the use of OM-PD, we can archive both high precision and long-term stabilisation performance. The ultra-low timing jitter proves that the optical pulses have been extremely well stabilised to the microwave reference.

### Experimental setup for an ultra-stable optical-pulse-train distribution system with fibre stabilisation

**Figure 2:**
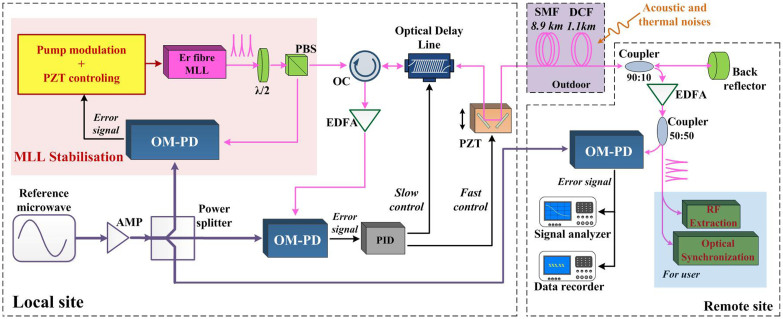
Experimental setup for the optical-pulse-train distribution and measurement system. A stabilised optical pulse train is distributed to a “remote site” via a 10-km fibre link. At the remote site, a portion of the pulse train is designated for the user. The rest is reflected back to the “local site” via the same fibre link. An optical-microwave phase-detection technique is used to generate a high-precision phase-error signal to compensate for the phase fluctuations in the fibre link.

The experimental setup for our distribution system with fibre stabilisation is illustrated in Figure 2. A classical round-trip fibre-stabilisation method is used here to compensate for the phase fluctuation induced by acoustic and thermal noise. A 6-GHz microwave signal generated by a commercial RF signal generator (Agilent, E8257D) is used as the reference microwave. Using an RF signal amplifier and a 1-to-3 power splitter, we obtain three identical microwave reference signals with powers of ~25 mW at the local site. One of the microwave signals is used for the stabilisation of the MLL via the OM-PD. The second signal is used to measure the phase fluctuation of the fibre link. The third is used for the out-of-loop measurement. The laser beam generated by the MLL is split into two beams. One beam, with a power of ~10 mW, is phase detected using a reference microwave in an OM-PD to generate an error signal for the phase locking of the MLL. The other beam, with a power of ~30 mW, is fed into a fibre link. The fibre link consists of a motorised optical delay line (ODL), a fast PZT-based optical delay line, a 1.1-km dispersion-compensated fibre (DCF) and an 8.9-km commercial single-mode fibre (SMF).The fibre link is installed in spools and directly exposed to an outdoor environment. The total attenuation of the fibre link is ~7 dB. The optical pulses received at the remote site have durations of less than 100 ps. A 90:10 fibre coupler and a back reflector are used to cause 90% of the received optical beam to be reflected back, whereas the remaining10% of the beam is used for RF signal extraction and measurements. The portion of this beam with lower power first passes through an Er-doped fibre amplifier (EDFA) to compensate for the optical loss from the 90:10 fibre coupler. The optical pulses then pass through a 50:50 fibre coupler. One portion of the beam, with a power of ~27 mW, is used for an out-of-loop performance test that determines the phase difference between the optical pulses and the microwave reference in an out-of-loop OM-PD, whereas the remainder is available to the user for high-accuracy RF signal extraction or optical-optical synchronisation^31^,^32^,^33^,^34^,^35^.

The 90% of the optical pulse trains that are reflected are received by an optical circulator at the local site. A second EDFA is also used at this location to obtain high-power pulses. The backward pulses are amplified to ~25 mW and fed into an OM-PD to detect the phase fluctuation for a single round trip. The phase-error signal is filtered by a proportional-integral-derivative (PID) module to remove long-term drift and fast noise. Then, the PID-regulated error signal is used to drive the PZT-based optical delay line at a high rate (~1000 times/s), whereas the motorised ODL is adjusted at a rate of ~1 time/s to ensure that the fibre length drift is within the locking bandwidth of the PZT-based optical delay.

### Frequency instability and phase noise of the optical-pulse-train distribution

**Figure 3:**
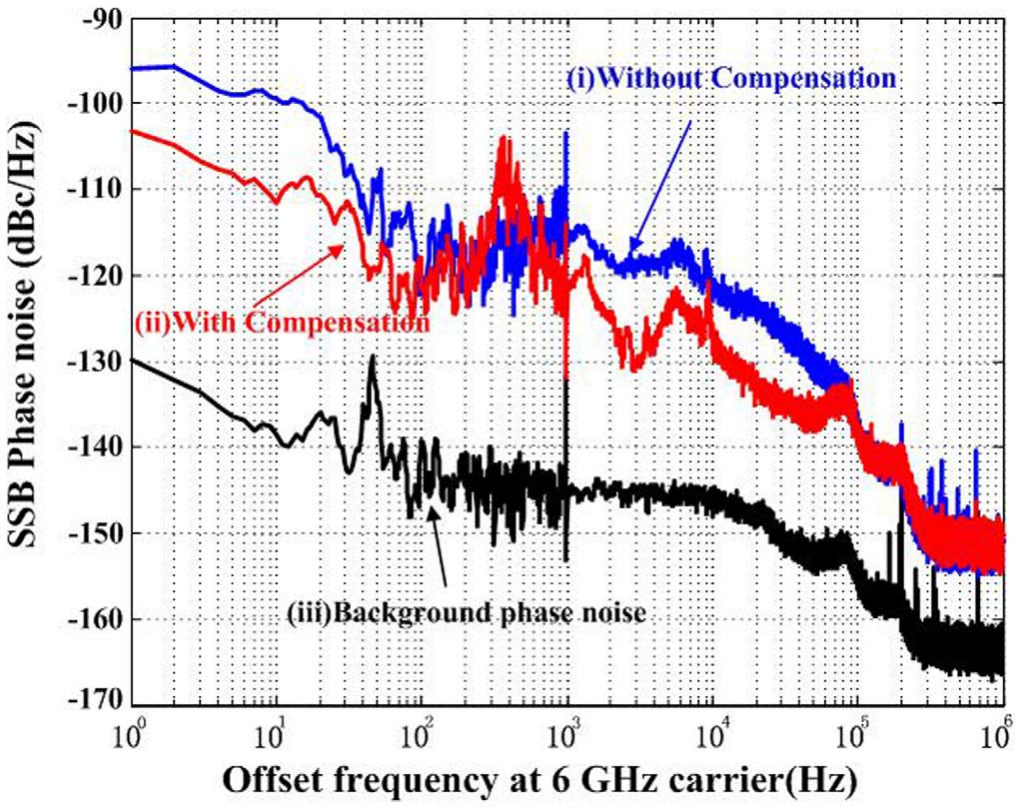
Out-of-loop residual phase noise at the remote site. (i) Out-of-loop residual phase noise of the optical-pulse-train distribution system without link compensation. (ii) Out-of-loop residual phase noise of the optical-pulse-train distribution system with compensation (integrated rms timing jitter of 3.8 fs [1 Hz to 100 kHz]). (iii) Background noise of the OM-PD.

**Figure 4:**
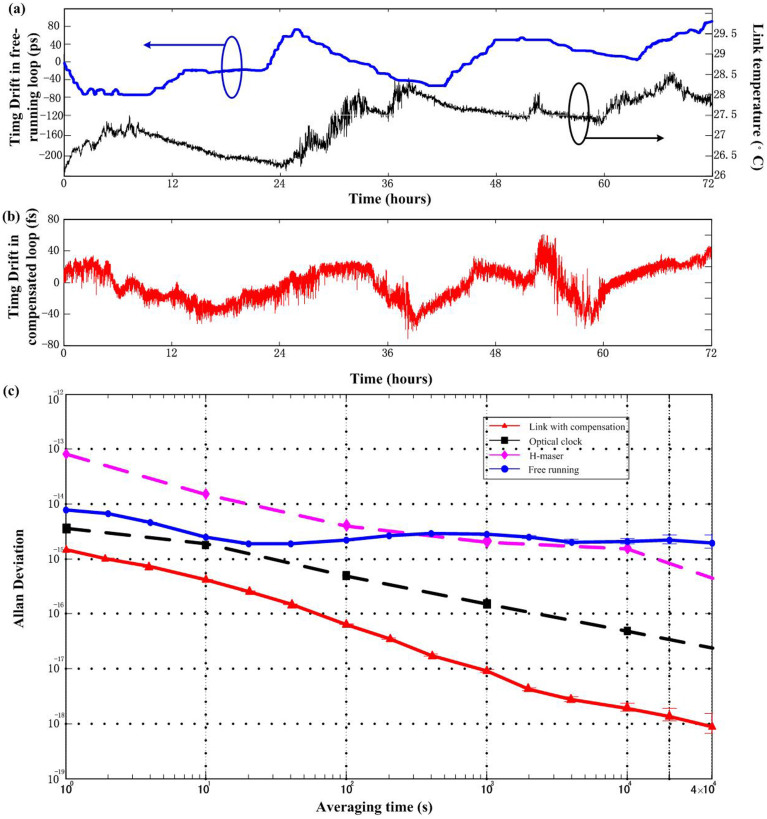
Residual instability of the optical-pulse-train distribution system. (a) Timing drift of the optical-pulse-train distribution system without compensation (rms timing drift of 44 ps over 72 hours) and temperature change. (b) Timing drift of the optical-pulse-train distribution system with compensation (rms timing drift of 22 fs over 72 hours). (c) The measured fractional frequency instabilities. The blue line (filled circles) is the result for the free-running fibre link, the red line (filled triangles) is the result with phase-fluctuation compensation, and the pink line (filled diamonds) and black line (filled squares) represent the frequency stabilities of the H-maser clock and a Sr optical clock, respectively, which are presented for comparison.

Figure 3 presents the phase-noise performance of the optical-pulse-train distribution measured at the out-of-loop. As in most studies, the residual phase noise of the distributed pulse timing signal is measured to determine the short-term distribution performance. Figure 3 shows that the phase noise with link compensation reaches -104 dBc/Hz and -140 dBc/Hz at 1 Hz and 100 kHz offset frequency, respectively. By integrating the phase noise in a bandwidth of 1 Hz to 100 kHz, we find a short-term integrated timing jitter of 3.8 fs. The timing jitter reaches sub-10-fs scale. We also test the long-term performance of our system by recording the residual timing drift. Figure 4a presents the timing drift in free-running loop and fibre link temperature change over a 72-hour period of operation. Figure 4b presents the timing drift in compensated loop. The drift is measured and calculated using a separate OM-PD and a high-accuracy voltmeter (3-Hz measurement bandwidth, 2 samples/s). A 22-fs rms timing drift is observed, while the drift range could achieve as high as 160-ps (peak to peak) (40-ps rms) when the compensation system is turned off. Furthermore, the Allan deviation calculated from the recorded timing-drift data represents the residual instability of the distributed pulse timing signal (filled triangles in Figure 4c). Figure 4c shows that the instability reaches 2.0 × 10^−15^ and 8.8 × 10^−19^ for averaging times of 1 s and 40,000 s, respectively (3-Hz measurement bandwidth). This residual instability is even lower than that of the H maser clock^36^ or an optical clock^37^. The demonstrated technique in our study can be potentially applicable to more and more stable Sr optical clock.

## Discussion

In this study, we demonstrate a high-precision fibre-based optical-pulse-train distribution system. In the system, we remove traditional devices, such as photodiodes and microwave mixers, because photodiodes can introduce shot noise, thermal noise and AM-to-PM noise. Instead, we apply several OM-PDs to perform the tasks of MLL stabilisation and fibre-link stabilisation. The OM-PD is constructed based on the principle of the Sagnac loop and is very robust and reliable. Different with the research presented in Ref. 33, we use a different ultra-stability MLL and a new stabilization method to make sure ultra-low noise RF can be received at remote end. Compared with Ref. 21 and 22, we archive a better performance of phase noise and long-term transfer instability. Especially for phase noise and short-term stability, the instabilities are improved by over one order of magnitude.The timing-stabilised pulse trains at the end-station have many applications; for example, they can be used to synchronise with a VCO for RF extraction or an MLL for ultra-low-noise optical-optical synchronisation. Many alternative electro-optic techniques have been demonstrated for RF extraction^31^,^32^,^34^,^35^ and optical-optical synchronisation^2^,^33^. All these techniques have reached the sub-fs level, which is sufficient for signal synchronisation.

The results of the experiment prove that our study provide a ultra-low-noise system for the distribution of optical pulse signals which can achieve accuracy at sub-10-fs scale for short-term performance (1 s) and at 30-fs scale for long-term performance (72 hours) for long-distance (longer than 10 km) transfer via a low-cost SMF link, while the sub-5-fs integrated timing jitter could also be achieved. Compared with other high-accuracy fibre-stabilisation techniques, *e.g.*, the use of periodically poled KTiOPO_4_ (PPKTP) single-crystal cross-correlators^2^,^33^, the OM-PD method is much more easily implemented and more robust, with a larger timing-delay detection range and a lower cost.

The demonstrated residual instability of our distribution system is superior to that of optical clocks. This makes it possible to synchronise the timing signal of an optical clock to a remote facility when the optical pulses are stabilised to the optical clock. Especially, with further improvement, the proposed frequency-distribution technique may provide a very powerful tool for transferring the timing signals of most stable Sr optical clocks without loss of stability. Compared with Ref. 40, we did longer distance and lower noise optical-pulse-train distribution, which is more suitable for that of optical clocks. Meanwhile, the system is potentially suitable for use in timing synchronisation for the next generation of XFELs and particle accelerators, various parts of which may need to be distantly located to achieve sufficient accelerating distance while working in tightly synchronised conditions. These facilities are being built throughout the world, although the technologies to satisfy their requirements of extremely high timing-synchronisation accuracy are still under development. The demonstrated technique which can provide sub-10-fs accuracy in short term and 10^−19^ level in long term is a potential tool for most facilities, while the 10-km distribution distance is also long enough for them. Furthermore, the different harmonics of the pulse trains' repetition rate can be simultaneously extracted into multiple RF signals with different frequencies. This is a valuable feature for microwave extraction at various frequencies. Similar with the system in Ref. 40, the performance of our system is also limited by polarization mode dispersion. In future studies, the techniques proposed in this work could be applied to the distribution of time and frequency signals over free space to reduce the effect.

## Methods

### The Er-doped fibre MLL

We use a passively nonlinear polarisation rotation (NPR) mode-locked Er-doped fibre as the optical source. The MLL operates in the stretched-pulse regime and has a fundamental repetition rate of 173 MHz. Its 35^th^ harmonic of repetition rate, 6 GHz, is used for RF signal extraction and phase measurements to provide a high sensitivity. A 40-cm highly doped Er gain fibre with a cumulative anomalous group-velocity dispersion is used, and the remainder of the fibre cavity is constructed using common SMF. To reduce the intra-cavity negative dispersion, we use a space isolator instead of a fibre isolator. By optimising the ratio of the positive dispersion fibre to the negative dispersion fibre, we set the intra-cavity dispersion to the close-to-zero dispersion condition to minimise the timing jitter. The MLL is pumped through a 980-nm/1550-nm wavelength-division multiplexing (WDM) fibre coupler by a 650-mW, 980-nm diode. A polarisation beam splitter, a half-wave plate and two quarter-wave plates are used to induce the mode locking. The output power of the MLL is ~50 mW.

### Description of optical-microwave phase detectors (OM-PDs)

A detailed description of the OM-PD can be found in Ref. 34. It was originally proposed in 2004 (Ref. 38) and demonstrated in 2012 (Ref. 34) by Jungwon Kim, and the authors tested its application for link-stabilisation purposes. The OM-PD fundamentally performs the phase detection based on the theory of Sagnac-loop interferometery^39^. The OM-PD consists of a circulator, a unidirectional high-speed LiNbO_3_ phase modulator and a specially designed polarisation-maintaining (PM) fibre Sagnac loop of a short length. When a microwave signal with a frequency that is an integer multiple of that of the laser is applied to the unidirectional phase modulator, the copropagating pulses experience a phase modulation, whereas the counterpropagating pulses do not. The phase of the copropagating pulses is modulated according to the temporal position between the optical pulses and the driving microwave signals. The power difference between the two outputs of the Sagnac loop is proportional to the phase error between the optical pulse trains and the driving microwave signal. A balanced photodetector is used for precise optical-microwave phase detection. The most important feature of the OM-PD is that the implementation of phase detection is completed optically prior to photodetection.

### Stabilisation of an MLL via both pump modulation and PZT control

The stabilisation of our MLL is based on both pump modulation and PZT control. The principle behind the use of pump modulation to adjust the phase of the MLL is that a change in the intra-cavity optical intensity will lead to linear changes in the refraction indices of both the Er-doped fibre and the SMF. The underlying basis of this principle is that the intra-cavity optics will interact with the Er atoms and the silicon in the cavity based on nonlinear effects, and these effects occur in such a manner that when the optical intensity changes linearly over a very short range, the refraction indices of the two materials will also change linearly^28^. The repetition rate *f_r_* of the MLL is determined by the MLL cavity length *L* and the average refractive index of the cavity *n* as follows: 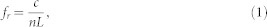
where *c* is the speed of light. It can be observed that changing *n* will also change *f_r_*, thereby changing the phase of the laser. Although the range of adjustment is short, the advantage of the pump-modulation method is that the modulation speed and accuracy are greatly improved compared with the use of a PZT alone^28^.

Therefore, to achieve long-term and high-accuracy stabilisation, we combine pump modulation and PZT control. Whereas pump modulation is used to achieve fast, high-accuracy stabilisation, long-term stabilisation is ensured by means of adjusting the PZT length. A commercial current supply (Thorlabs, ITC110) is used for the pump modulation, and the PZT (PI, P-840.20) is driven by a 100-V voltage supply and has an adjustment range of 30 μm.

### Fibre-link stabilisation using both fast and slow feedbacks

We use both motorised and PZT-based optical delay lines to stabilise the fibre link. The advantage of this method is that the accuracy is very high, and the compensation range is also very large. The PZT-based optical delay line (XMT, 40VS12) is driven by a 150-V voltage supply and has a resolution of 2 fs/V and a compensation range of 200 fs. The motorised optical delay line (General Photonics, MDL-002) has an adjustment accuracy of 1 fs and a large compensation range of 500 ps. It is driven slowly to ensure that the fast drift remains within the compensation range of the PZT-based optical delay line. The stabilisation link and the long fibre link operate in a reciprocal manner, so by simply locking the phase error between the local microwave reference and the backward optical pulse trains to nearly zero, the entire link can be stabilised, ensuring that optical pulses with stable phases are received at the remote site. Furthermore, phase fluctuations induced by optical-intensity noise can be significantly reduced by detecting the near-zero phase differences, as in this way the detections are operated when the in-Sagnac-loop signals are almost orthogonal.

## Author Contributions

J.Y.Z. and B.N. developed the concept. J.T.W. designed the mode-locked laser, Z.B.L. designed the fibre link, B.N. and S.Y.Z. designed the OM-PDs, B.N., D.H. and J.Y.Z. designed the RF signal-processing circuits and other electronic servo systems. S.Y.Z. collected the data. All authors joined in the discussion and provided comments. B.N. and S.Y.Z. contributed equally to this work.
